# Cutaneous amyloidosis as the first presentation of Waldenstrom macroglobulinemia

**DOI:** 10.22088/cjim.11.3.340

**Published:** 2020-05

**Authors:** Rana Rafiei, Hojat Eftekhari, Behnam Rafiee

**Affiliations:** 1 Skin Research Center, Department of Dermatology, Razi Hospital, School of Medicine, Guilan University of Medical Sciences, Rasht, Iran; 2 Department of Pathology, NYU Winthrop Hospital, 222 Station Plaza, No. 620, Mineola, NY 11501, USA

**Keywords:** Primary systemic amyloidosis, Waldenstrom macroglobulinemia

## Abstract

**Background:**

Waldenstrom macroglobulinemia is a lymphoplasmacytic lymphoma with elevated serum immunoglobulin M and multi-organ involvement. Primary systemic amyloidosis usually develops due to immunoglobulin light chains depositions in different organs due to an underlying gammopathy.

**Case presentation:**

Our patient was an 86-year-old man with macroglossia, ecchymotic patches and bullous lesions associated with a skin laxity on the periorbital, palmar, and glans penis areas. Skin biopsy confirmed dermal amyloid depositions. In serum immunofixation electrophoresis, prominent monoclonal immunoglobulin-M lambda light chains were detected associated with prominent lymphoplasmacytic infiltration in bone marrow biopsy which was diagnosed as Waldenstrom macroglobulinemia.

**Conclusion:**

Skin involvement presenting as cutaneous amyloidosis could be the first manifestation of Waldenstrom macroglobulinemia. We should think about an underlying gammopathy in an old patient with skin laxity and ecchymosis.

Primary systemic amyloidosis (PsA) is a clinical presentation of plasma cell dyscrasia which does not meet the criteria of multiple myeloma because it does not have hypercalcemia or bone lesions. It usually develops due to immunoglobulin light chain depositions in different organs such as oral cavity, skin, kidney, heart, liver, bone marrow and neural system (1). PsA characteristically presents by easy bruising especially in periorbital areas, macroglossia and carpal tunnel syndrome (2, 3). Skin lesions have been clinically detected in almost 25% of these cases (1). Herein we present an 86-year-old man with a history of recurrent ecchymotic lesions and skin laxity on the glans penis and fingertips since a year ago. 

## Case presentation

**Figure 1:**
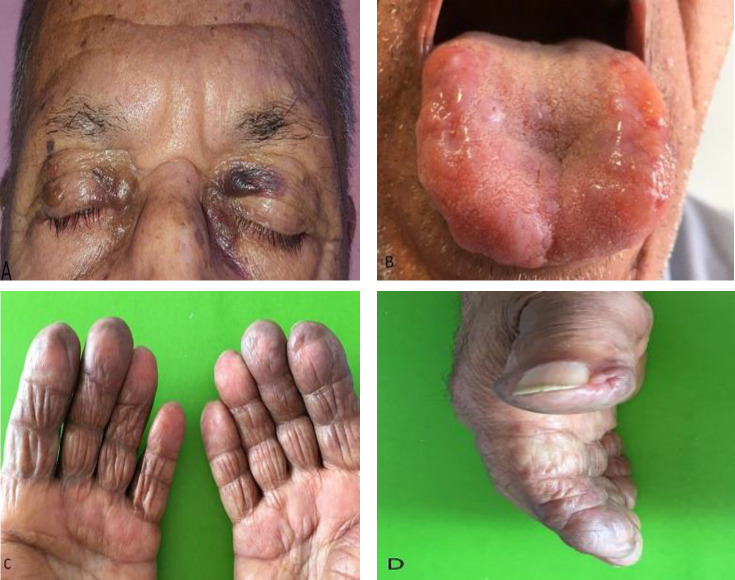
Some ecchymotic patches and bullous lesions are seen on the periorbital areas (A). Macrglossia with papulonodular lesions (B). Fingertips are ecchymotic, loose, soft, and redundant (C). Some waxy papules are seen on the nail fold (D).

An 86-year-old farmer man with a one-year history of recurrent ecchymotic lesions and puffiness on the fingertips and glans penis was referred to our outpatient clinic. Also, he mentioned that periorbital bullous lesions developed during the last four months and acral numbness associated with difficult swallowing, dizziness, sweating and constipation appeared during last three months. There was no history of weight loss, fatigue, dyspnea or hoarseness. On physical examination these findings were detected: His fingertips were ecchymotic with a loose, soft, and redundant skin which remained depressed for more than 3 minutes after applying pressure. Some ecchymotic patches and bullous lesions were seen on the periorbital, palmar, and glans penis areas. Also a large and firm tongue with papulonodular lesions and some waxy papules on the proximal nail folds of two fingers were detected (figure 1a,b,c,d).

**Figure 2:**
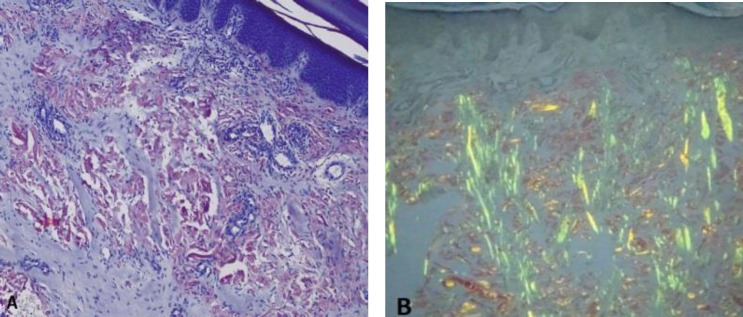
Depositions of amorphous eosinophilic material are seen throughout dermis, positively stained with Congo red (A) with apple-green birefringence under polarized light evaluation (B). (Congo red staining, original magnification × 100)

There was no organomegaly or lymphadenopathy. Past medical history included diabetes, hypertension and prostatic problems, so he was under treatment with glibenclamide, nifedipine and tamsulosin. Skin biopsy was done from right palmar lesions and left thumb papules which showed depositions of amorphous eosinophilic material throughout dermis, positively stained with Congo red with apple-green birefringence under polarized light evaluation (figure 2 a,b). 

Crystal violet staining also was positive. Laboratory work-up were as follows: hemoglobin 10.6 g/dL, erythrocyte sedimentation rate 22 mm/h, blood urea nitrogen 33 mg/dl (reference, 7-21), creatinin 1.47 mg/dl (reference, 0.7-1.4), lactate dehydrogenase 487 U/L (reference, 230-460), serum albumin 4.4 g/dL (reference, 3.5-5), and total protein of 6.5 g/dL (reference, 6.6-8.3). Leukocyte and platelet count, liver and thyroid function tests, coagulation tests, serum calcium, phosphorus, ferritin, and C-reactive protein were within normal limit.There was 1+ proteinuria in urine analysis and 1368 mg protein in 24-hour urine collection test. Serum Immunofixation electrophoresis showed prominent monoclonal immunoglobulin-M lambda light chains, 291.94 mg/L (reference, 5.71-26.30). Chest and skull imaging were normal. Ultrasound revealed a simple cyst in the left kidney and lower limit size of both kidneys but no organomegaly was detected. Echocardiogram showed mild left ventricular hypertrophy, normal right ventricular function, mild regurgitation of mitral, tricuspid, and aortic valves with normal cardiac output. Brain CT-scan showed no specific finding. Physical examinations and neurologic evaluations using nerve conduction velocity test, confirmed bilateral carpal tunnel syndrome. Bone marrow biopsy showed trabeculae and intervening marrow with 70-80% cellularity diffusely infiltrated by small to medium sized lymphocytes. Immunohistochemistry study showed diffusely CD20 and CD138 positive lymphocytes with features of both lymphocytes and plasma cells (lymphoplasmacytic cells). These histopathologic features associated with serum electrophoresis findings were consistent with “lymphoplasmacytic lymphoma/ Waldenstrom macroglobulinemia (LPL/ WM)”. The patient was referred to hematologist/oncologist for appropriate treatment. He was on leukeran (chlorambucil) and prednisolone during eight months follow-up with a good general condition. Examination of the patient was conducted according to the Declaration of Helsinki principles.

## Discussion

Waldenstrom macroglobulinemia (WM) is a lymphoplasmacytic lymphoma with elevated serum immunoglobulin M and multi-organ involvement. WM is usually seen in old men with non-specific symptoms including anorexia, weight loss, and weakness. Peripheral neuropathy and Raynaud’s phenomenon may be the first manifestations. It has been defined as an IgM monoclonal gammopathy with >10% bone marrow infiltration by CD20^+^ small lymphocytes which exhibit plasmacytoid or plasma cell differentiation (CD138^+^). Systemic amyloidosis has been reported in 3% of WM cases which is mainly of the light chains type (4, 5). Amyloidosis develops due to extracellular deposition of several proteinaceous insoluble materials such as immunoglobulin (Ig) light chains (AL), Ig heavy chains (AH), amyloid A (AA), and beta2-microglobulin in different organs (1). Systemic amyloidosis has different subtypes including primary, secondary, and hereditary. PsA could be idiopathic or myeloma-associated and develops due to AL aggregations. Secondary systemic amyloidosis usually occurs due to chronic inflammation and AA accumulations. Cutaneous involvement is rare in the secondary subtype but it is usually detected in the primary amyloidosis (1, 2). Skin lesions could be the presenting signs of AL amyloidosis in 20%-40% of patients (1, 3). Easy bruising on acral and periorbital areas associated with macroglossia could be valuable signs of an underlying gammopathy. Ecchymotic macule, especially in the periorbital region has been named as pinch purpura which develops after minor trauma due to skin fragility (1-3). Dermal amyloid deposits reduce flexibility of dermal connective tissue and increase fragility of the dermal vessels, so pinch purpura and hemorrhagic-bullous lesions may appear (3, 6). It seems that cutaneous flexibility and elasticity of the fingertips decreased in our patient which is similar to cutis laxa. It has been proposed that elastic tissue may be replaced or changed by amyloid depositions which results in skin laxity in patients with amyloidosis (6, 7). Acral paresthesia, dysphagia, dizziness, sweating and constipation in our patient could be due to neural involvement associated with AL amyloidosis (1-3).

The most common laboratory finding in patients with symptomatic WM is anemia, similar to our patient and could be explained by following etiologies: reduced survival of red blood cell, disturbed erythropoiesis, and hemolysis (8). In indolent or smoldering type of WM, there is no evidence of end-organ damage, such as constitutional symptoms, anemia, hyperviscosity, lymphadenopathy, or organomegaly which are usually seen in plasma cell proliferative disorders (7, 9). Progression of smoldering WM to symptomatic WM, amyloidosis, or lymphoma has been estimated 68% during 10 years. So close follow-up of smoldering WM patients is essential (9). Alkylating agents such as chlorambucil are suitable for initial treatment of WM in elderly patients. Treatment continues until a plateau state in disease progression then could be discontinued with close follow-up of patients (7).

In conclusion, we should always think about an underlying gammopathy in an old patient with skin laxity and ecchymosis.
